# Development of antibiotic treatment algorithms based on local ecology and respiratory surveillance cultures to restrict the use of broad-spectrum antimicrobial drugs in the treatment of hospital-acquired pneumonia in the intensive care unit: a retrospective analysis

**DOI:** 10.1186/cc13990

**Published:** 2014-07-15

**Authors:** Liesbet De Bus, Lies Saerens, Bram Gadeyne, Jerina Boelens, Geert Claeys, Jan J De Waele, Dominique D Benoit, Johan Decruyenaere, Pieter O Depuydt

**Affiliations:** 1 Department of Critical Care Medicine, Ghent University Hospital, Ghent, Belgium; 2 Department of Information Technology, Ghent University iMinds, Ghent, Belgium; 3 Department of Laboratory Medicine, Ghent University Hospital, Ghent, Belgium; 4 Heymans Institute of Pharmacology, Ghent University, Ghent, Belgium

## Abstract

**Introduction:**

Timely administration of appropriate antibiotic therapy has been shown to improve outcome in hospital-acquired pneumonia (HAP). Empirical treatment guidelines tailored to local ecology have been advocated in antibiotic stewardship programs. We compared a local ecology based algorithm (LEBA) to a surveillance culture based algorithm (SCBA) in terms of appropriate coverage and spectrum of antimicrobial activity.

**Methods:**

We retrospectively assessed 2 hypothetical empirical antibiotic treatment algorithms for HAP on an existing high-quality prospectively collected database in a mixed 36-bed tertiary intensive care unit (ICU). Data on consecutive episodes of microbiologically confirmed HAP were collected over a period of 40 months and divided in a derivation (1 July 2009 to 31 October 2010) and validation (1 November 2010 until 31 October 2012) cohort. On the derivation cohort we constructed a LEBA, based on overall observed bacterial resistance patterns, and a SCBA, which targeted therapy to surveillance culture (SC) in the individual patient. Therapy was directed against pathogens found in respiratory SC collected two to five days before HAP, and in the absence of these, presence or absence of multi-drug resistant (MDR) pathogens in other SC dictated broad-spectrum, respectively narrow spectrum antibiotic therapy. Subsequently, LEBA and SCBA were retrospectively reviewed and compared with actually prescribed antibiotics in the validation cohort.

**Results:**

The first 100 HAP episodes made up the derivation cohort and the subsequent 113 HAP episodes the validation cohort. Appropriate antibiotic coverage rates by applying LEBA and SCBA were 88.5% and 87.6%, respectively, and did not differ significantly with respect to appropriateness of the actually prescribed initial therapy (84.1%). SCBA proposed more narrow spectrum therapy as compared to LEBA and the actually prescribed antimicrobials (*P* <0.001). SCBA recommended significantly less combination therapy and carbapenems compared to LEBA (*P* <0.001). SCBA targeted antibiotics to recent respiratory SC in 38.1% (43 out of 113 episodes) of HAP; in these cases adequacy was 93% (40 out of 43).

**Conclusion:**

Rates of appropriate antimicrobial coverage were identical in LEBA and SCBA. However, in this setting of moderate MDR prevalence, the use of SCBA would result in a significant reduction of the use of broad-spectrum drugs and may be a preferential strategy when implementing antibiotic stewardship programs.

## Introduction

Antibiotic stewardship refers to efforts both made to improve appropriateness of antibiotic prescription and to reduce antibiotic selection pressure by limiting unnecessary use of antibiotics, especially those with a broad spectrum [1,2]. As hospital-acquired pneumonia (HAP) is a frequent indication for antibiotic prescription as well as a manifestation of antibiotic resistance, antibiotic policy for HAP is an important target area for antibiotic stewardship. Early appropriate antibiotic therapy is a major determinant of outcome in HAP: *early* refers usually to the time of the initial clinical diagnosis or suspicion of pneumonia [3-5]. As at this early stage, microbial etiology is still unknown and potentially multi-drug resistant (MDR), broad-spectrum antibiotics, often in combination schemes, are advocated as empirical therapy. As the microbial and resistance patterns are variable across ICUs, these empirical schemes have to be matched to the local situation in order to achieve high rates of appropriate coverage whilst avoiding unnecessary broad-spectrum antibiotics [6,7]. In addition, algorithms may contribute to antibiotic stewardship as they assist to rationalize antibiotic choices and reduce prescription variability, improve overall appropriateness and restrain use of certain drug classes such as carbapenems. As a more controversial approach, early antibiotic therapy may be guided by surveillance cultures (SC) to improve its appropriateness [8-11]. With this approach, antibiotics are essentially selected in order to cover colonizing pathogens in the individual patient.

In this study, we developed two algorithms for initial antibiotic prescription in ICU patients with suspected HAP. We aimed a) to assess the potential of an algorithm to aid in antibiotic stewardship in our setting and b) quantify the contribution of SC to antibiotic stewardship as compared to empirical therapy based upon local epidemiology.

## Materials and methods

### Clinical setting and design

This retrospective analysis was conducted at the 14-bed Medical ICU and the 22-bed Surgical ICU of the Ghent University Hospital (1,056 beds). With the aid of the software application, Computer-based Surveillance and Alerting of infections, Antimicrobial Resistance and Antibiotic consumption in the ICU (COSARA), all episodes of pneumonia were registered prospectively from 1 July 2009 to 31 October 2012. COSARA assists the attending ICU-physician in acquiring an overview of the various daily collected data related to infection diagnosis (trends in laboratory values, temperature, oxygenation et cetera) and treatment. This includes a graphical display of current and past antibiotic treatments as a timeline and provides direct links to a real-time copy of the various source records. The graphical interface allows the user to label infectious episodes during daily clinical rounds and interdisciplinary staff meetings to a predefined list of diagnoses in all patients admitted to the ICU. As such, the program facilitates the build-up of an extensive data-warehouse on antibiotic use and infection in the ICU [12]. During the study period treating physicians were not guided in the choice of the empirical antimicrobial by treatment algorithms. The Ghent University Hospital ethics committee approved the study and waived informed consent as prospective registration did not affect treatment decisions, and all subsequent analyses were performed retrospectively on an anonymized database. Only patients aged 16 years or above were included.

### Definition of hospital-acquired pneumonia

Pneumonia was defined to be hospital-acquired if it occurred 48 h or more after admission to the hospital. HAP was defined clinically as the presence of new and/or progressive and persistent pulmonary infiltrates on the chest radiograph, in combination with two or more of the following criteria: worsening of oxygenation, increase in purulent tracheobronchial secretions, presence of fever (≥38.5°C) or hypothermia (≤36°C). Only microbiologically confirmed HAP was included: confirmation consisted of the isolation of a respiratory pathogen with at least 1+ semiquantitative growth of a good quality respiratory sample (defined as <3 squamous epithelial cells per low-power field) obtained within one calendar day prior or after clinical diagnosis of HAP. In our hospital, microbiological analysis of respiratory samples routinely consists of semiquantitative culture of endotracheal aspirate (ETA) in the ventilated patient or sputum in the non-intubated patient. For logistic reasons, broncho-alveolar lavage (BAL) is not systematically performed, similarly to current practice in the majority of European ICUs [13]. We previously found that BAL and ETA had good qualitative and quantitative concordance in a cohort of patients with suspected ventilator-associated pneumonia (VAP). Positive and negative predictive values of a semiquantitative growth score of 1+ of a pathogen in ETA to identify the same pathogen in a quantity of at least 10^4^ colony-forming units (CFU)/ml in BAL were 81% and 87%, respectively [14]. HAP was defined to be ventilator-associated if at the time of diagnosis, patients were under mechanical ventilation for 48 h or longer, or had been extubated for less than 48 h after mechanical ventilation for at least 2 days.

### Development of the algorithms

**Figure 1:**
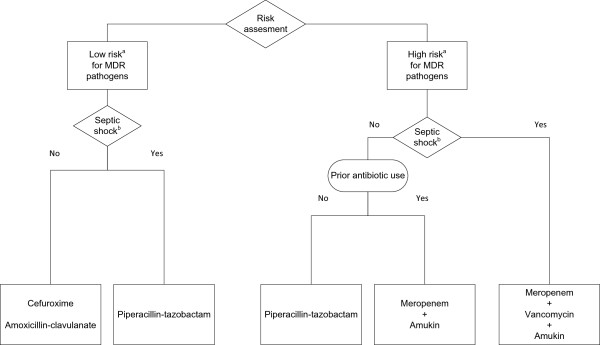
**Local ecology-based algorithm. **^a^Clinical risk assessment for multi-drug resistant pathogens: high risk if one of the following characteristics is present: prior antimicrobial therapy; current hospitalization ≥ 5 days; hospitalization for ≥2 days in the preceding 6 months. ^b^Septic shock was defined as systolic arterial blood pressure <90 mmHg or mean arterial blood pressure <65 mmHg despite adequate fluid resuscitation. MDR, multi-drug resistant.

**Figure 2:**
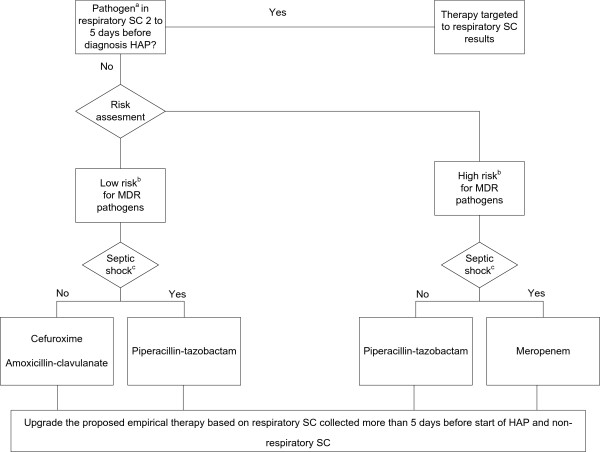
**Surveillance culture-based algorithm. **^a^Respiratory pathogen defined as: *Acinetobacter spp.*, Enterobacteriaceae, *Haemophilus spp*., *Pseudomonas spp.*, *Staphylococcus aureus*, *Stenotrophomonas spp*., Streptococci. ^b^Clinical risk assessment for multi-drug resistant pathogens: high risk if one of the following characteristics is present: prior antimicrobial therapy; current hospitalization ≥5 days; hospitalization for ≥2 days in the preceding 6 months. ^c^Septic shock was defined as systolic arterial blood pressure <90 mmHg or mean arterial blood pressure <65 mmHg despite adequate fluid resuscitation. SC, surveillance cultures; HAP, hospital-acquired pneumonia; MDR, multi-drug resistant.

The collected data were divided into a derivation and a validation cohort. The first 100 HAP episodes (1 July 2009 to 31 October 2010) made up the derivation cohort for the development of the local ecology-based algorithm (LEBA) and the surveillance culture-based algorithm (SCBA), both aiming to achieve a minimum of 85% appropriate coverage rate. For LEBA (Figure 1), we started from the clinical framework of the revised American Thoracic Society-Infectious Diseases Society of America (ATS-IDSA) guidelines and our previously recorded antimicrobial resistance patterns [4]. Clinical risk factors for MDR pathogens were defined as prior antimicrobial therapy during the current hospitalization, a hospital stay of 5 days or more, and previous hospitalization for 2 days or more in the preceding 6 months. SCBA (Figure 2) combined the same clinical risk factors for MDR with microbiological information from systematically collected SC. The SC consisted of oral, nasal and rectal swabs and urinary cultures upon admission, followed by thrice-weekly urinary and once-weekly oral, nasal and rectal samples in all patients, as well as thrice-weekly sputum in the non-intubated patient or ETA in the ventilated patient. In the case of positive respiratory SC (oral swabs or respiratory samples) 2 to 5 days before diagnosis of HAP, the antibiotic with the narrowest spectrum possible covering this (these) pathogen(s) was proposed (see also Table 1). In the absence of these, an alternative algorithm was proposed guided by clinical risk factors as in LEBA, but with upgrading to include all pathogens isolated from other SC collected within the last 6 months (respiratory SC more than 5 days before HAP and non-respiratory SC) (see also Figure 2). Both algorithms were retrospectively reviewed and compared with the actually prescribed antimicrobial therapy in the validation cohort.

**Table 1:** **Scale quantifying the spectrum of the antibiotic treatment**

**Step**	**Treatment**
1	Non-antipseudomonal penicillins (amoxicillin-clavulanate)
	Second generation or third generation non-antipseudomonal cephalosporins (cefuroxime, ceftriaxone)
	Trimethoprim/sulfamethoxazole
2	Antipseudomonal penicillins (piperacillin-tazobactam)
	Third generation antipseudomonal cephalosporins (ceftazidime)
3	Fluoroquinolones (ciprofloxacin, levofloxacin, moxifloxacin)
4	Antipseudomonal carbapenems (meropenem)
5	Combination therapy of two or more antibiotic agents

### Appropriateness and spectrum of antimicrobial therapy

We compared rates of appropriateness and spectrum between LEBA, SCBA and actually prescribed antimicrobial therapy by the treating physician. Therapy was considered appropriate when all pathogens involved in the HAP episode were covered by the antibiotic, or by at least one component of the antibiotic combination. To quantify the antimicrobial spectrum, we constructed a scale ranging from 1 - the most narrow-spectrum of empirical therapy, lacking antipseudomonal activity - to 5 - a combination therapy of two or more antibiotic agents (Table 1). We ranked fluoroquinolones higher than broad spectrum antipseudomonal β-lactam antibiotics other than carbapemens, based upon the knowledge that exposure to fluoroquinolones is particularly associated with rapid emergence of MDR pathogens such as methicillin-resistant *Staphylococcus aureus* (MRSA) [15], and with Clostridium difficile-associated diarrhea [16], and by our aim to preserve the use of fluoroquinolones for directed therapy of *Stenotrophomonas* spp. and *Pseudomonas aeruginosa*. In case of appropriate therapy, the antimicrobial spectrum was expressed as *x steps in excess* to the most narrow-spectrum therapy possible covering all causative pathogens isolated in the HAP episodes.

### Statistics

Continuous variables are described as mean (±standard deviation) or median (interquartile range) for normal or non-normal distribution, respectively. To compare paired proportions the McNemar test for related samples was used. Differences in medians were checked using the Wilcoxon signed-rank test. All statistical analyses were performed with SPSS® software (SPSS, version 21, Chicago, IL, USA). Statistical significance was defined as *P* <0.05.

## Results

All data reported apply to the validation cohort of 113 episodes of HAP, including 52 (46%) episodes of VAP, registered between 1 November 2010 and 31 October 2012 in 104 patients. There was need for subsequent mechanical ventilation in 39/61 (64%) of the non-VAP patients. The median age of the patients was 64 years (54 to 74), and 74% were male. In 99 (87.6%) of the HAP episodes, clinical risk factors for MDR pathogens were present: prior antibiotics in 81%, current hospitalization for 5 days or more or hospitalization in the previous 6 months in 82%. Septic shock was present in 23% of the HAP episodes. The length of ICU stay following diagnosis of HAP was 10 days (6 to 22) when appropriate antibiotics were administered, 7 days (2 to 16) if the prescribed antibiotics were inappropriate (*P* = 0.10). The overall ICU mortality was 30.1% and did not differ between patients with or without appropriate antimicrobial therapy (28.4% versus 38.9%, *P* = 0.375).

A total of 140 pathogens were isolated, 84% of which were Gram-negative bacteria (Table 2). HAP was mono-microbial in 89 (79%) and poly-microbial in 24 (21%) episodes.

**Table 2:** Pathogens (n = 140) associated with HAP

**Pathogen**	**Number of total**
**Gram-positive bacteria**	
*Staphylococcus aureus*	14 (10.0)
MRSA	(5%)
*Streptococcus pneumonia*	5 (3.6)
Other streptococci	1 (0.7)
**Gram-negative bacteria**	
**Enterobacteriaceae**	**70 (50.0)**
*Escherichia coli*	31 (22.1)
*Enterobacter sp.*	13 (9.3)
*Klebsiella sp.*	12 (8.6)
*Serratia sp.*	6 (4.3)
*Morganella morganii*	4 (2.9)
*Citrobacter sp.*	2 (1.4)
*Hafnia alvei*	1 (0.7)
*Proteus sp.*	1 (0.7)
ESBL-producing enterobacteriaceae	(5.7%)
**Non-fermenters**	**35 (25)**
*Pseudomonas aeruginosa*	27 (19.3)
Ceftazidim resistance	(5%)
Carbapenem resistance	(6.4%)
*Stenotrophomonas maltophilia*	5 (3.6)
Acinetobacter baumannii*	3 (2.1)
**Other gram-negative bacteria**	
*Haemophilus influenzae*	12 (8.6)
*Moraxella catarrhalis*	3 (2.1)
**Total**	140

*All *Acinetobacter baumannii* isolates were third generation cephalosporin-resistant and carbapenem sensitive. HAP, hospital-acquired pneumonia; MRSA, methicillin-resistant *Staphylococcus aureus*; ESBL, extended-spectrum beta-lactamase producing enterobacteriaceae.

### Appropriateness and spectrum of antimicrobial therapy

Appropriate antibiotic therapy was prescribed in 95 (84.1%) HAP episodes. Antimicrobial choices proposed by LEBA and SCBA were appropriate in 88.5% and 87.6%, respectively. Paired analysis showed no significant difference in adequacy for the different strategies (prescribed therapy versus LEBA: *P* = 0.33; prescribed therapy versus SCBA: *P* = 0.5; LEBA versus SCBA: *P* = 0.99). Pathogens associated with inadequate empirical therapy are detailed in Table 3.

**Table 3:** Pathogens associated with inadequate empirical therapy

**Pathogen**	**Prescribed therapy**	**LEBA**	**SCBA**
*Acinetobacter baumannii*	3	-	1
*Escherichia coli*	4	-	2
*Enterobacter sp.*	2	-	-
*Klebsiella sp.*	-	-	1
MRSA	-	4	3
*Pseudomonas aeruginosa*	4	3	4
*Serratia sp.*	2	2	2
*Stenotrophomonas maltophilia*	3	5	2

Results are presented as number. LEBA, local ecology-based algorithm; SCBA, surveillance culture-based algorithm; MRSA, methicillin-resistant *Staphylococcus aureus.*

**Figure 3:**
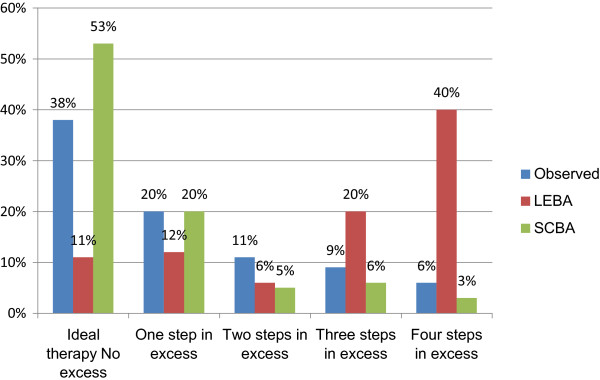
**Evaluation of the spectrum of antimicrobial therapy.** LEBA, local ecology-based algorithm; SCBA, surveillance culture-based algorithm. LEBA: 3 (1.25 to 4) steps in excess; SCBA: 0 (0 to 1) steps in excess; Prescribed therapy: 1 (0 to 2) steps in excess.

In significantly more episodes, SCBA proposed antibiotics of a narrower spectrum as compared to both the prescribed therapy and the regimen suggested by LEBA (*P* <0.001) (Figure 3). Significantly less combination therapy was proposed by SCBA (7.1%) in comparison with LEBA (81.4%) (*P* <0.001). SCBA recommended carbapenems in significantly fewer episodes than LEBA (24 (21.2%) versus 92 (81.4%), respectively (*P* <0.001)).

### Surveillance culture-based algorithm

Respiratory SC sampled 2 to 5 days before HAP onset were available in 63 episodes (55.8%) of HAP, of which 43 (68%) grew at least one pathogen. As such, SCBA suggested targeted antimicrobial therapy in 43/113 (38.1%) of HAP episodes: HAP for which targeted therapy was suggested was ventilator-associated in 72% (31/43), occurred more than 5 days after ICU admission in 77% (33/43) and was caused by the following pathogens (n = 53): *S. aureus* (6/53, 3 methicillin-susceptible *S. aureus* and 3 MRSA), *S. pneumonia* (1/53), Enterobactereaceae (29/53), *P. aeruginosa* (10/53), other Gram-negative bacteria (7/53). Recent respiratory SC accurately predicted all causative pathogens in 81.4% (35/43) of HAP; SCBA-targeted antimicrobial therapy would appropriately cover all causative pathogens (including those not predicted by SC) in 93% (40/43) of cases.

In the case of negative or absent respiratory SC 2 to 5 days before start of HAP (n = 70, 61.9%), SCBA took into account both respiratory SC more than 5 days prior to infection and non-respiratory SC. In 28/70 (40%) of these HAP episodes positive SC were available, leading to upgrading of the proposed empirical therapy in 13/70 HAP (19%) and a switch from inappropriate to appropriate antibiotic proposals in 10 episodes. By not upgrading our therapy in these cases our rate of appropriate antibiotic therapy would have dropped from 87.6% to 78.8%.

## Discussion

Both guidance by SC as well as the use of ICU-specific empirical schemes that incorporate local microbiology data have been shown to increase appropriate empirical prescription and reduce the use of broad-spectrum antimicrobials as compared to general guidelines [4,7,8,17-19]. Our study is the first to demonstrate the benefit of SC in surplus to tailoring guidelines to local susceptibility data. We found that incorporating results of SC (SCBA) in a clinical algorithm (LEBA) to help the choice of an empirical antibiotic regimen in suspected HAP would allow reduction in the use of broad-spectrum antimicrobials for equal rates of appropriate coverage. In particular, a 60% decrease in the empirical use of carbapenems would be attained, which is an important achievement in terms of antibiotic stewardship. Similarly, as compared to actually prescribed antibiotics, which were at the discretion of the attending physician with access to SC results but without guidance by a treatment algorithm, stricter adherence to SCBA would lead to further constraint of empirical use of broad-spectrum drugs. We measured the expenditure of antibiotics in terms of extension of spectrum by ranking antimicrobial classes along a scale of increasingly broad antimicrobial coverage. While this scale artificially translates a complex phenomenon into a simplified score, it allows some quantification of ecological selection pressure between different antibiotic schemes.

Two observations underlie the construction of SCBA. First, previously we found high negative predictive values of negative SC for the presence of MDR pathogens in HAP [9], allowing a narrower-spectrum antibiotic even in patients with clinical risk factors for MDR. Second, we followed the paradigm that ICU-acquired pneumonia is often preceded by colonization of the upper and lower airways by the same pathogen, going through a possible intermediate stage of ventilator-associated tracheobronchitis [20]. Following Bayes’ theorem, the positive and negative predictive values of SC were then applied to the ATS-IDSA guideline-based clinical risk categories for MDR HAP. Although there are no reports suggesting resistant micro-organisms cause more septic shock, we opted for broader therapy in these cases to minimize the risk of harm caused by inappropriate therapy.

In hospital-acquired infection, narrowing the spectrum of antibiotic therapy is usually done as de-escalation following an initially broad-spectrum therapy aimed at maximal chance for appropriate coverage. However, limiting antimicrobial therapy upfront may offer several advantages. First, aminoglycosides and glycopeptides, which carry an important toxicity profile [21,22], were abandoned in the SCBA if there were no SC results supporting their need. A study in patients with pneumonia found increased mortality in patients who were treated with strict adherence to the ATS-IDSA guideline, including the recommendations for combination therapy, as compared to patients in whom treatment deviated from the recommendations [23]. The authors proposed the toxic effects of combination antimicrobial therapy as a potential explanation. Second, although prolonged exposure to antibiotic therapy has been clearly associated with the emergence of resistance [24], there is no proof that a short course is ecologically harmless and devoid of selection pressure. Finally, there exists a gap between the concept of de-escalation and what is achieved in practice. In several observational studies, the authors found rates of de-escalation to be fairly low [25-27], with lack of an identifiable microbial agent as the main barrier. SCBA partially circumvents this, as in case of negative diagnostic cultures and SC, a narrower spectrum empirical therapy, is recommended as compared to LEBA.

Restricting the number of empirical combination therapies will reduce direct antibiotic costs. On the other hand one fulltime-equivalent microbiology laboratory technician is assigned to process SC of all patients admitted to our 36-bed ICU and the cost for the laboratory material is estimated at 33 euro per week. However, not all of this cost is exclusively for surveillance purposes, as few additional respiratory cultures for diagnostic purposes are required under this SC regime. Additionally, it can be argued that SC are a cornerstone in infection control in settings where MDR pathogens are endemic and that their guidance of antibiotic therapy is only an added benefit [28,29].

A number of limitations have to be addressed. First, our study is evidently monocentric and our SCBA is site-specific. However, the concept of SCBA may be more universally applicable, as the predictive values of SC as reported in several recent studies are fairly consistent, provided that SC are regularly and at least twice weekly sampled [19]. Our SCBA could serve as a template, which has to be translated into antibiotic recommendations depending on local ecology and carefully assessed before implementation. Second, resistance rates are moderate in our setting and the added value of SC in combination with guidelines tailored to local susceptibility data has to be re-evaluated in settings with higher resistance rates. As targeted antimicrobial therapy was proposed by SCBA in more than one third of HAP episodes, we suspect that implementing this algorithm would also lead to reduction in empirical broad-spectrum combination antibiotic therapy in these high-resistance environments. Third, it would be safe to regularly test the algorithms in order to match potentially changing ecology. Fourth, this analysis was performed retrospectively, subsequently the algorithms and adherence by the treating physicians to the algorithms have not been evaluated in practice. As such, the performance of the algorithms may in reality be different from what is anticipated. Finally, our study design does not allow us to conclude whether an empirical strategy with de-escalation, as compared to a strategy that is more targeted to colonizing pathogens translates into a different patient outcome or microbiological selection pressure.

## Conclusion

As compared to an algorithm based upon clinical risk factors for MDR and adapted to local susceptibility results, an algorithm with additional guidance from SC could achieve comparably high rates of appropriate coverage with the use of fewer broad-spectrum antibiotics. Antibiotic therapy specifically targeted to respiratory pathogens identified in recent SC would be possible in 38% of HAP episodes. SC-guided algorithms may constitute a component of antibiotic stewardship programs. Additional studies should be performed in ICU settings with higher levels of antibiotic resistance.

## Key messages

• Addition of surveillance culture results in empirical antibiotic treatment algorithms for hospital-acquired pneumonia could restrict the use of broad-spectrum antimicrobial drugs.

• Targeting empirical treatment to recent respiratory surveillance cultures could be achieved in more than one third of hospital-acquired pneumonia.

## Abbreviations

ATS-IDSA: American Thoracic Society-Infectious Diseases Society of America; BAL: broncho-alveolar lavage; CFU: colony forming units; COSARA: Computer-based Surveillance and Alerting of infections, Antimicrobial Resistance and Antibiotic consumption in the ICU; ETA: endotracheal aspirate; HAP: hospital-acquired pneumonia; LEBA: local ecology-based algorithm; MDR: multi-drug resistant; MSSA: methicillin-susceptible *Staphylococcus aureus*; MRSA: methicillin-resistant *Staphylococcus aureus*; SC: surveillance cultures; SCBA: surveillance culture-based algorithm; VAP: ventilator-associated pneumonia.

## Competing interests

The authors declare that they have no competing interests.

## Authors’ contributions

LDB conceived, designed and coordinated the study, performed data acquisition and analyses and drafted the manuscript, LS performed data acquisition and analyses and critically revised the manuscript for important intellectual content, BG contributed to data acquisition and analyses and critically revised the manuscript for important intellectual content, JB contributed to data acquisition and analyses and critically revised the manuscript for important intellectual content, GC contributed to data acquisition and analyses and critically revised the manuscript for important intellectual content, JDW contributed to data acquisition and analyses and critically revised the manuscript for important intellectual content, DB contributed to data acquisition and analyses and critically revised the manuscript for important intellectual content, JD contributed to data acquisition and analyses and critically revised the manuscript for important intellectual content, PD conceived, designed and coordinated the study, performed data acquisition and analyses and drafted the manuscript. All authors read and approved the final manuscript. All authors agree to be fully accountable for the content of this work.
